# Supplementary material for: Peer reviews of peer reviews: A randomized controlled trial and other experiments
Source: PLoS One. 2025 Apr 2;20(4):e0320444. doi: 10.1371/journal.pone.0320444 (PMC11964232; doi:10.1371/journal.pone.0320444)
Supplement: S1 Appendix — Questionnaire for participants (PDF) [file pone.0320444.s001.pdf]

Questionnaire for participants

In this section, we present the questionnaires given to the participants in our study to evaluate paper reviews both on overall quality and on four specific criteria.

Provide an overall score for the quality of the review:

1. **Very low** E.g., a generic review that is applicable to any paper / a short and dismissive review.
2. **Low** E.g., a review with serious flaws on multiple aspects.
3. **Fair** E.g., a review with serious flaws on one aspect / a review without serious flaws but with limited insights for Authors/Area Chairs.
4. **Good** E.g., acceptable review, but nothing stands out. Moderately helpful for decision-making.
5. **Very good** E.g., a helpful review that stands out on some aspects and provides useful insights.
6. **Excellent** E.g., a very insightful review that stands out on all aspects.
7. **Exceptional** E.g., an excellent review that helps authors to non-trivially improve the paper / brings a unique piece of information that is crucial for the decision.

Agree or disagree with the following statements (5-item Likert):

1. The review demonstrates an adequate understanding of the paper.
   - Review makes comments that are detailed and specific to the paper
   - It is OK for the reviewer to lack expertise in certain aspects of the paper as long as it is explicitly or implicitly indicated in the review
2. The review covers all the required aspects.
   - Review adequately comments on Soundness, Presentation, and Contribution of the paper
   - Review adequately comments on Strengths and Weaknesses of the paper
3. Evaluations made in the review are well supported.
   - Objective arguments are grounded in the paper's content (e.g., specific results/comparisons) and are correct
   - Subjective arguments are accompanied with reasoning
   - A review that brings additional useful information (e.g., counter examples or uncited references which do a part of the claimed work) is especially strong
4. The review provides constructive feedback to authors.
   - Whenever possible, critical comments (especially subjective) are accompanied with actionable items on how to improve the paper
   - Review is unbiased and written in a polite manner
