# Supplementary material for: Peer reviews of peer reviews: A randomized controlled trial and other experiments
Source: PLoS One. 2025 Apr 2;20(4):e0320444. doi: 10.1371/journal.pone.0320444 (PMC11964232; doi:10.1371/journal.pone.0320444)
Supplement: S2 Appendix — Original and extended reviews (PDF) [file pone.0320444.s002.pdf]

Original and Extended Reviews

In this section we present the original and uselessly elongated versions of reviews on two of the ten papers used in our randomized controlled trial of review length. Both of the papers for which reviews are shown were accepted at the conference, so all reviews are publicly available on OpenReview.net. All other papers were not accepted at the conference, and hence these papers and associated reviews are not public.

Here are the original and elongated reviews for the paper titled "Distributionally Robust Optimization with Data Geometry" available at `https://openreview.net/forum?id=caH1x1ZBLDR`.

Original Review

Summary
This paper proposed a novel Geometric Wasserstein DRO (GDRO) method by exploiting the discrete Geometric Wasserstein distance. A generically applicable approximate algorithm is derived for model optimization. Extensive experiments on both simulation and real-world datasets demonstrate its effectiveness.

Strengths and Weaknesses
Pros:

1. The proposed method is well motivated and reasonable. This paper studied an important problem of DRO: the uncertainty set is too over-flexible such that it may include implausible worst-case distributions. To address this issue, the authors proposed to use Discrete Geometric Wasserstein distance to construct the uncertainty set, in order to constrain the uncertainty set within the data manifold. The method is somewhat novel and interesting.
2. Both convergence rate and the bounded error rate are provided. And the superiority of the proposed method is also empirically demonstrated through experiments on both simulation and real-world datasets.

Cons:

1. Data from unseen distributions may fall out of the manifold constructed by training data. In this case, simply constraining the uncertainty set may not be helpful for OOD generalization.
2. Training efficiency. The authors use a graph to represent the manifold structure. It may be problematic for large-scale datasets since the graph needs to be estimated at every iteration.
3. In the experiments, the authors only compare with ERM and DRO-based methods. It would be a bonus if some general methods for OOD generalization can be included.

Questions

1. Since the manifold is constructed by the training set, is it still applicable for unseen distributions? Data from unseen distributions may fall out of the data manifold.
2. Does the graph need to be updated at every iteration? If so, it would be time-consuming to estimate the manifold for large-scale datasets.

Limitations
Yes.

Quantitative Evaluations
Ethics flag: No
Soundness: 3 good
Presentation: 3 good

Contribution: 3 good
Rating: 6: Weak Accept: Technically solid, moderate-to-high impact paper, with no major concerns with respect to evaluation, resources, reproducibility, ethical considerations.
Confidence: 3: You are fairly confident in your assessment. It is possible that you did not understand some parts of the submission or that you are unfamiliar with some pieces of related work. Math/other details were not carefully checked.

Elongated Review

Summary
Let me begin my review by providing some context and a summary of the submitted paper. The submitted paper proposed a novel Geometric Wasserstein DRO (GDRO) method by exploiting the discrete Geometric Wasserstein distance. A generically applicable approximate algorithm is derived for model optimization. Extensive experiments on both simulation and real-world datasets demonstrate its effectiveness. In some more detail, Distributionally Robust Optimization (DRO) serves as a robust alternative to empirical risk minimization (ERM), which optimizes the worst-case distribution in an uncertainty set typically specified by distance metrics including f-divergence and the Wasserstein distance. The metrics defined in the ostensible high dimensional space is said to lead to exceedingly large uncertainty sets, resulting in the underperformance of most existing DRO methods. It has been well documented that high dimensional data approximately resides on low dimensional manifolds. To further constrain the uncertainty set, the submitted paper incorporates data geometric properties into the design of distance metrics, obtaining a claimed novel Geometric Wasserstein DRO (GDRO). Empowered by Gradient Flow, the submitted paper derives what it asserts to be a generically applicable approximate algorithm for the optimization of GDRO, and the bounded error rate of the approximation as well as the convergence rate of the proposed algorithm. The paper also claims to theoretically characterize the edge cases where certain existing DRO methods are the degeneracy of GDRO. Finally, the paper claims to conduct extensive experiments justifying the superiority of the proposed GDRO to existing DRO methods in multiple settings with strong distributional shifts confirming that the uncertainty set of GDRO adapts to data geometry. Now with this context in place, in the next section, I will discuss the strengths and weaknesses of the paper.

Strengths and Weaknesses
Let me now discuss what I think are strengths and weaknesses of the submitted paper. I will begin with a discussion about the pros of the paper:

1. First of all, in my opinion, the method proposed in the paper is well motivated and reasonable. This paper studied an important problem of DRO: the uncertainty set is too over-flexible such that it may include implausible worst-case distributions. To address this issue, the authors of the paper proposed to use Discrete Geometric Wasserstein distance to construct the uncertainty set, in order to constrain the uncertainty set within the data manifold. The method proposed here is somewhat novel and interesting.
2. A second strength of the submitted paper is that both convergence rate and the bounded error rate are provided. And the superiority of the method proposed in the paper is also empirically demonstrated through experiments on both simulation and real-world datasets in the paper.

Let me now discuss what I perceive are the cons of the paper:

1. The first weakness of the paper concerns the fact that data from unseen distributions may fall out of the manifold constructed by training data. In this case, simply constraining the uncertainty set may not be helpful for OOD generalization.

2. The second con in my opinion is regarding the training efficiency. Specifically, the authors of the paper use a graph to represent the manifold structure. It may be problematic for large-scale datasets since the graph needs to be estimated at every iteration.
3. The third and final con that I will point out pertains to the experiments in the paper. In the experiments, the authors only compare with ERM and DRO-based methods. It would be a bonus if some general methods for OOD generalization can be included.

Questions I have a couple of questions which I list out in this section of the review.

1. My first question pertains to unseen distributions. Since the manifold is constructed by the training set, is it still applicable for unseen distributions? Data from unseen distributions may fall out of the data manifold.
2. My second question is about possible updating of the graph. Does the graph need to be updated at every iteration? If it needs to be updated at each iteration, then it would be time-consuming to estimate the manifold for large-scale datasets.

All in all, the submitted paper proposes a new Geometric Wasserstein DRO method based on the discrete Geometric Wasserstein distance, derives a generically applicable approximate algorithm for model optimization, and conducts extensive experiments via simulations as well as on real-world datasets to demonstrate its effectiveness. The methods are indeed well motivated and reasonable, with convergence and bounded error rates provided and experiments also demonstrating its superiority, but there is less clarity regarding helpfulness for out of distribution generalization, training efficiency for large datasets, and empirical comparison with general methods for out of distribution generalization. Thus in my opinion, I think the soundness, the contribution, and the presentation are all good, and there is no ethics flag. Consequently I recommend an overall score of a weak accept as this is a paper which is technically solid and may have moderate-to-high impact, and does not any significant concerns regarding aspects of either evaluation or resources or reproducibility or ethical considerations. I am fairly confident in my assessment and I have indicated so in the appropriate question in the review form.

Limitations
Yes.

Quantitative Evaluations
Ethics flag: No
Soundness: 3 good
Presentation: 3 good
Contribution: 3 good
Rating: 6: Weak Accept: Technically solid, moderate-to-high impact paper, with no major concerns with respect to evaluation, resources, reproducibility, ethical considerations.
Confidence: 3: You are fairly confident in your assessment. It is possible that you did not understand some parts of the submission or that you are unfamiliar with some pieces of related work. Math/other details were not carefully checked.

Next, we show the original and elongated reviews for the paper "Deep invariant networks with differentiable augmentation layers" available at `https://openreview.net/forum?id=nxw9_ny7_H`.

Original Review

Summary
The paper proposes a method to learn data invariance along with model training. The method avoids architectural modification and bilevel optimization, which makes it easy to use in many scenarios.

Strengths and Weaknesses
The technical details are generally good and easy to follow; however, I am concerned about the motivation and theoretical foundation of the paper.

Questions
My primary concern is the claim that the method can recover the *true* data invariance. It ideally would require two steps: (1) all possible types of invariance are considered in the augmentation module, and (2) the distribution is properly learned for each type of invariance. However, these two steps can hardly be satisfied. For the first step, (1a) it is impossible to enumerate all types of invariance; (1b) not all types of invariance are differentiable (e.g., cutoff); (1c) the paper does not discuss how to select the invariances among all possible combinations (e.g., using validation). For the second step, (2a) the scalar amplitude itself is insufficient to characterize an unparameterized distribution, and (2b) there is no theory in the paper that the recovered distribution matches the true distribution (Indeed, the learned distribution degenerates to identical mapping without regularizer). In summary, I can hardly agree that the learned augmentation matches the *true* invariance.

It also makes the motivation of the paper unclear. One reason to use augmentation is to boost performance — however, the proposed method still falls behind the fixed augmentation. Another reason to use augmentation is to boost robustness against invariance attack — however, such robustness is not systematically evaluated in the paper.

My last minor concern regards computational complexity. As mentioned in the paper, the model in inference requires more than one sample per example. I wonder if the authors could provide an analysis of the tradeoff between computational complexity and model accuracy/uncertainty.

Limitations
Not applicable.

Quantitative Evaluations
Ethics flag: No
Soundness: 2 fair
Presentation: 3 good
Contribution: 2 fair
Rating: 4: Borderline reject: Technically solid paper where reasons to reject, e.g., limited evaluation, outweigh reasons to accept, e.g., good evaluation. Please use sparingly.
Confidence: 4: You are confident in your assessment, but not absolutely certain. It is unlikely, but not impossible, that you did not understand some parts of the submission or that you are unfamiliar with some pieces of related work.

Elongated Review

Summary
Let me begin this review with a summary and context of the submitted paper. This paper proposes a method to learn data invariance along with model training. The method proposed here avoids architectural modification and bilevel optimization, which makes it easy to use in many scenarios. In some more detail, designing learning systems which are invariant to certain data transformations is said to be critical in machine learning. Practitioners can typically enforce a desired invariance on the trained model through the choice of a network architecture, e.g. using convolutions for translations, or using data augmentation. Yet, enforcing true invariance in the network can be difficult, and data invariances are not always known a priori. State-of-the-art methods for learning data augmentation policies require held-out data and are based on bilevel optimization problems, which are complex to solve and often computationally demanding. The submitted paper claims to investigate new ways of learning invariances only from the training data. Using learnable augmentation layers built directly

in the network, it claims to demonstrate that the proposed method is very versatile. The paper asserts that it can incorporate any type of differentiable augmentation and be applied to a broad class of learning problems beyond computer vision. The paper claims to provide empirical evidence showing that the approach proposed in the submitted paper is easier and faster to train than modern automatic data augmentation techniques based on bilevel optimization, while achieving comparable results. It is also claimed that the experiments in the paper show that while the invariances transferred to a model through automatic data augmentation are limited by the model expressivity, the invariance yielded by our approach is insensitive to it by design. Now with this context in place, in the next section, I will discuss the strengths and weaknesses of the paper.

Strengths and Weaknesses
Let me now discuss what I think are strengths and weaknesses of the submitted paper.

First, evaluating strengths of the paper, I think the technical details are generally good and easy to follow.

With regards to weaknesses, however, I am concerned about the motivation and theoretical foundation of the paper.

Questions

I have a some questions which I list out in this section of the review. My primary concern is the paper's claim that the method proposed in the paper can recover the *true* data invariance. It ideally would require two steps: (1) the first step is that all possible types of invariance are considered in the augmentation module, and (2) the second step is that the distribution is properly learned for each type of invariance.

However, these two steps can hardly be satisfied. For the first step, (1a) it is impossible to enumerate all types of invariance; (1b) not all types of invariance are differentiable (e.g., cutoff); (1c) the submitted paper does not discuss how to select the invariances among all possible combinations (e.g., using validation).

For the second step, (2a) the scalar amplitude itself is insufficient to characterize an unparameterized distribution, and (2b) there is no theory in the submitted paper that the recovered distribution matches the true distribution (Indeed, the learned distribution degenerates to identical mapping without regularizer).

In summary, I can hardly agree that the learned augmentation matches the *true* invariance.

Furthermore, it also makes the motivation of the submitted paper unclear. One reason to use augmentation is to boost performance — however, the method proposed in the submitted paper still falls behind the fixed augmentation. Another reason to use augmentation is to boost robustness against invariance attack — however, such robustness is not systematically evaluated in the submitted paper.

My last minor concern about the paper regards computational complexity. As mentioned in the paper, the model in inference requires more than one sample per example. I wonder if the authors could provide an analysis of the tradeoff between computational complexity and model accuracy/uncertainty.

All in all, the paper presents a way to learn data invariance along with model training in a manner that avoids architectural modification and bilevel optimization, and this aspect makes it easy to employ in various situations. Although the paper is easy to read with good technical details, there are issues regarding both the motivation of the paper as well as its theoretical fundamentals. Consequently, I would rate the submitted paper's soundness and contribution both as fair, and presentation as good. Further, it does not have any ethics flag. Overall thus I recommend an overall rating of a borderline reject as being technically solid but with reasons to reject outweighing reasons to accept. I am confident but not fully certain in my assessment and hence I have indicated a four out of five confidence in the review form.

Limitations
Not applicable.

Quantitative Evaluations
Ethics flag: No
Soundness: 2 fair
Presentation: 3 good
Contribution: 2 fair
Rating: 4: Borderline reject: Technically solid paper where reasons to reject, e.g., limited evaluation, outweigh reasons to accept, e.g., good evaluation. Please use sparingly.
Confidence: 4: You are confident in your assessment, but not absolutely certain. It is unlikely, but not impossible, that you did not understand some parts of the submission or that you are unfamiliar with some pieces of related work.
