# Supplementary material for: Peer reviews of peer reviews: A randomized controlled trial and other experiments
Source: PLoS One. 2025 Apr 2;20(4):e0320444. doi: 10.1371/journal.pone.0320444 (PMC11964232; doi:10.1371/journal.pone.0320444)
Supplement: Attachment — Submitted filename: response_to_reviewers.pdf [file pone.0320444.s003.pdf]

Response to Reviewers

We thank the two reviewers for their thoughtful and detailed comments. We also thank the academic editor Guglielmo Campus for the prompt handling of the paper. In this document, we provide an itemized response to the reviewers' comments. We have also submitted a revised version of our paper where our changes are marked in red colored text.

Reviewer 1

1. *Section 3.1.1. Methods repeats some information that's already in section 2.1.*

   We have removed repeated description of the high-level experimental setup from Section 3.1.1, since this is included in 2.1. Now, section 2.1 focuses on the statistical methodology of the hypothesis test, while 2.1 describes the overall experimental setup.

2. *The number of evaluators and evaluations recruited is impressive, and given that there were no incentives, surprising. It would help to add some detail on the recruitment pipeline – was the questionnaire and consent step implemented during the course of the normal review process? And the RCT probably happened later? Or perhaps this information is already there and I missed it?*

   We have added this context as well as details on the recruitment pipeline and overall timeline of the experiment in Section 2 (Experimental Setup.) We note that NeurIPS is quite a large conference—there were over 9,000 paper submissions and 15,000 registered attendees of the conference in the 2022 conference at which we conducted the experiment. For the reviewer's convenience, we reproduce the newly added text below:

   "Recruitment occurred after paper submission, during the review period, but before final accept/reject decisions were released. Specifically, we recruited the (meta)-reviewers and authors from July 11 to August 11, 2022 and external reviewers from August 11 to August 25, 2022. The conference used the OpenReview.net conference management platform for the review process. Reviewers, meta-reviewers, and authors were sent a notification about the experiment via this platform, asking if they would like to opt-in."

   "For context, at the conference there were 824 meta-reviewers, 10,406 reviewers and 9,634 paper submissions in total."

   "They accessed the reviews through a cloud storage link (personalized for each evaluator) where the reviews and papers were temporarily stored and later deleted. They submitted their evaluations of review quality through a Qualtrics form. The evaluations were completed after paper reviews were completed and shown to authors, but before final decisions on paper acceptance and rejections were made."

3. *It would help giving a little more detail on the choice/setup of the Mann-Whitney tests: for example, why are we calculating the confidence intervals and p-values separately, and using different number of iterations? And why the conventional Mann-Whitney or Wilcoxan rank-sum tests not appropriate here?*

   We are computing a standard Mann-Whitney U statistic (stratified across the 10 papers) and using a permutation test to determine statistical significance. We stratify across the 10 papers in order to control for any differences in the quality of reviews between papers. We separately bootstrap confidence intervals to be able to provide a range of values of the effect size in addition to a p-value. In our revision, we use the same number of iterations for the bootstrapped CI and the permutation test, although this did not change the results.

4. *I was a confused in section 3.2.1. criterion (iii). This suggests you wanted "consensus" papers? And how does this information from non-authors enter the test statistic? I expected some quantity like "Author score - mean(Non-authors core)" but didn't see it.*

We have added explicit descriptions of the purpose for each criteria in Section 3.2.1, reproduced below for the reviewer's convenience. Note that the criteria show up in the test statistic in that we only consider pairs of reviews (x_{accept}, x_{reject}) that meet criteria (i)-(iii).

Here are the descriptions of the purpose for each criteria that we added in the revision: "Criteria (i) controls for differences in evaluation of review quality between different authors or papers. Criteria (ii) controls for bias in evaluations due to length of reviews, as found in our RCT described in Section 3.1. Finally, criteria (iii) controls for underlying differences in the true review quality of the paired reviews. Since, we do not know the ground truth quality of a paper review, the evaluations from non-authors serve as a proxy for true quality. We restrict our analysis to reviews where the non-author evaluators agree in their quality evaluation."

5. *For the 3.5 Subjectivity analysis, commensuration bias, or in general differences in mapping are certainly one type of subjectivity, but other types exist too. In fact, it wasn't clear why this approach, estimating the loss, was more information than looking at the linear model of section 3.4.1. Why couldn't we take the variance of b_j and ϵ_i, j as the more natural definition of "subjectivity"? Furthermore, I don't think it's guaranteed that a final score is the result of *only* the criterion scores. What if the final score is some mapping from the criterion scores *and some unobserved criterion*, and let's say there is some subjectivity around that one – then we might have a consistent mapping function but still substantial loss.*

   We have made clearer in Section 3.5 that we are studying one specific type of subjectivity, changing the title of the section to "Subjectivity (commensuration bias)." We agree with the reviewer that the loss of the mapping from criteria scores to overall scores may be due to unobserved criteria in addition to commensuration bias. We have noted this as a limitation of the approach in our methods section and are not aware of methods that try to control for other latent sources of subjectivity in addition to commensuration bias.

   Additionally, we note that Section 3.4 (Miscalibration) and Section 3.5 (Subjectivity (commensuration bias)) aim to measure two distinct sources of arbitrariness in the review process—miscalibration captures differences in how evaluators judge the levels of the evaluation scale, while commensuration bias captures differences in what aspects of a paper review the evaluators consider important to overall review quality.

6. *It would help the reader contextualize the results if the abstract noted that responses weren't incentivized.*

   We have added the context that participation was voluntary in the abstract and that reviewers were not compensated to participate in the Section 2 (Experimental methods).

7. *If I understood correctly, the data availability statement is of the "Available upon request" type, but at the same time notes all the reasons why it can't be available. There even seems to be a promise to the participants that "The data will not be distributed," perhaps not even with anonymization. I really hope *something* can be made available publicly, as plenty of research has revealed that "available upon request" generally means "not available" and because I'm a big fan of the paper. It seems that thorough anonymization is quite possible here so in theory it should be possible to share the anonymized data. However if the promise made to participants is that nothing would be shared no matter what, then I think there's a challenge for PLOS policies.*

   We thank the reviewer for their positive comments and interest in the paper. In the consent forms of the study, we guaranteed the participants that "The data will not be distributed, and only aggregate findings may be published."

   Given this, we would appreciate guidance from the journal editor and reviewers regarding the best way to move forward in providing the maximum data transparency while being compliant with the data privacy protections guaranteed to study participants in the consent form.

Reviewer 2

1. *Please follow the journal's Vancouver style of formatting references.*

   We have corrected the reference style throughout to follow the Vancouver style of formatting references.

2. *How were the evaluators recruited? Were the email addresses obtained from the conference organizer or was there some other way? Similarly, how did the evaluators who agreed to participate receive the reviews?*

   We recruited evaluators directly through the OpenReview.net platform on which the conference was run. Four of this paper's authors were conference organizers (program chairs) of NeurIPS 2022.

   We have added the following details on recruitment to Section 2 (Experimental methods):

   "Participation was voluntary and no 129 compensation was given. Recruitment occurred after paper submission, during the review period, but before final accept/reject decisions were released. Specifically, we recruited the (meta)-reviewers and authors from July 11 to August 11, 2022 and external reviewers from August 11 to August 25, 2022. The conference used the OpenReview.net conference management platform for the review process. Reviewers, meta-reviewers, and authors were sent a notification about the experiment via this platform, asking if they would like to opt-in."

   We have added the following details on how evaluators received reviews and completed evaluations:

   "Evaluators were provided the review, along with the paper for which the review was written via email. They accessed the reviews through a cloud storage link (personalized for each evaluator) where the reviews and papers were temporarily stored and later deleted. They submitted their evaluations of review quality through a Qualtrics form. The evaluations were completed after paper reviews were completed and shown to authors, but before final decisions on paper acceptance and rejections were made."

3. *Since the authors chose certain questions from the RQI, and tailored the questions intended for machine learning, did the authors consider it a limitation that re-validation of the modified RQI would be warranted? The RQI was intended for medicine, so are the modified questions of the RQI questionnaire in this study measuring what they intend to measure?*

   Similar to prior work that develops and assesses the RQI [1, 2], we use multiple evaluators for many of the evaluations and report inter-reviewer consistency in Section 3.3 (Inter-evaluator (dis)agreement.) In addition, we note that our paper aims to understand the impact of biases, miscalibration, and subjectivity on the evaluations made using the RQI criteria. We find across all the questions asked that evaluations of the criteria can be impacted by length bias and author outcome bias, suggesting that asking these more concrete questions (in addition to a generic overall quality question) does not counteract the effect of these biases. Additionally, we reduced the number of questions in the RQI criteria as we chose to to prioritize brevity of survey questions in order to encourage completion of the survey among the broad set of untrained evaluators we recruited.

Thank you for your consideration,
Alexander Goldberg, Ivan Stelmakh, Kyunghyun Cho, Alice Oh, Alekh Agarwal, Danielle Belgrave, and Nihar B. Shah

References

[1] Van Rooyen S, Black N, Godlee F. Development of the review quality instrument (RQI) for assessing peer reviews of manuscripts. Journal of clinical epidemiology. 1999;52(7):625–629.

[2] Pranić SM, Malički M, Marušić SL, Mehmani B, Marušić A. Is the quality of reviews reflected in editors' and authors' satisfaction with peer review? A cross-sectional study in 12 journals across four research fields. Learned Publishing. 2021;34(2):187–197.
